# You Can’t Find Healthy Food in the Bush: Poor Accessibility, Availability and Adequacy of Food in Rural Australia

**DOI:** 10.3390/ijerph15102316

**Published:** 2018-10-21

**Authors:** Jill Whelan, Lynne Millar, Colin Bell, Cherie Russell, Felicity Grainger, Steven Allender, Penelope Love

**Affiliations:** 1 School of Medicine, Global Obesity Centre, Deakin University, Geelong 3220, Australia; colin.bell@deakin.edu.au; 2 Australian Health Policy Collaboration, Victoria University, Melbourne 3000, Australia; lynne.millar@vu.edu.au; 3 Australian Institute for Musculoskeletal Science (AIMSS), The University of Melbourne and Western Health, St Albans 3021, Australia; 4 School of Exercise and Nutrition Sciences, Deakin University, Geelong 3220, Australia; caru@deakin.edu.au (C.R.); fgrainge@deakin.edu.au (F.G.); penny.love@deakin.edu.au (P.L.); 5 School of Health and Social Development, Global Obesity Centre, Deakin University, Geelong 3220, Australia; steven.allender@deakin.edu.au; 6 Institute for Physical Activity and Nutrition (IPAN), Deakin University, Geelong 3220, Australia

**Keywords:** rural, food supply, food security, obesity

## Abstract

In high-income countries, obesity disproportionately affects those from disadvantaged and rural areas. Poor diet is a modifiable risk factor for obesity and the food environment a primary driver of poor diet. In rural and disadvantaged communities, it is harder to access affordable and nutritious food, affecting both food insecurity and the health of rural residents. This paper aims to describe the food environment in a rural Australian community (approx. 7000 km^2^ in size) to inform the development of community-relevant food supply interventions. We conducted a census audit of the food environment (ground truthing) of a local government area (LGA). We used the Nutrition Environment Measurement tools (NEMS-S and NEMS-R) to identify availability of a range of food and non-alcoholic beverages, the relative price of a healthy compared to a less healthy option of a similar food type (e.g., bread), the quality of fresh produce and any in-store nutrition promotion. Thirty-eight food retail outlets operated at the time of our study and all were included, 11 food stores (NEMS-S) and 27 food service outlets (NEMS-R). The mean NEMS-S score for all food stores was 21/54 points (39%) and mean NEMS-R score for all food service outlets was 3/23 points (13%); indicative of limited healthier options at relatively higher prices. It is difficult to buy healthy food beyond the supermarkets and one (of seven) cafés across the LGA. Residents demonstrate strong loyalty to local food outlets, providing scope to work with this existing infrastructure to positively impact poor diet and improve food security.

## 1. Introduction

Globally, obesity is a leading cause of chronic disease and premature death [1]. In low and middle-income countries, it impacts the wealthy, shifting to the rural poor as the country’s economy develops [1,2]. In high income countries, such as Australia, it impacts everyone but disproportionately affects those from more disadvantaged and rural areas [2]. Consequently, rural residents in Australia have higher rates of obesity and are more likely to die early from concomitant conditions including diabetes, heart disease and other chronic diseases [2,3].

Internationally and within Australia, poor diet has been identified as a leading modifiable risk factor contributing to the high burden of disease and obesity [3,4], and there is some evidence that the food environment is related to healthiness of diet among adults [5] and children [6]. These reviews highlight the lack of consistency in measurements of these associations which is to be expected in this emerging field but, nonetheless, some relationships are evident.

Some studies have identified that neighbourhoods without supermarkets have more diet-related health outcomes such as obesity and chronic disease [7]. In rural and/or disadvantaged communities, it is harder than in urban environments to access affordable and nutritious foods [8,9]. Availability varies as it is potentially constrained by the long distance required to travel to food stores [10], and limitations to fresh food supply delivery and increased prices occur with greater distance from the key metropolitan centres [11]. A consequence of these food supply constraints is that rural residents, and rural food retailers, in order to reduce the risk of waste, tend to purchase longer shelf-life foods [12], many of which may be less nutritious than the fresh options. Rural areas have a higher proportion of general stores to larger supermarkets compared to urban areas, providing fewer healthy options at higher prices and unpredictable quality [13,14]. These differences are most commonly attributed to limited transportation, storage and economies of scale for food distribution to rural areas [15].

Many rural areas experience diminishing population sizes, which reduces financial viability leading to the consolidation or closure of food stores [16,17]. With poor access to healthy food within close proximity, rural residents are reliant on transportation (public or private), incurring additional costs, and they frequently are forced to shop outside their local area, a phenomenon known as ‘out-shopping’ [17]. Out-shopping creates a vicious cycle for rural economies as revenue shifts to outside enterprises, and local businesses struggle to provide sufficient variety at low cost to attract and retain customers [17], adding further to rural economic decline. General stores, and ‘take-away’ food outlets, located in close proximity to residents, often with extended operating hours, may become the main source of food for rural communities [18], particularly those with limited mobility.

The food environment is defined as the “accessibility, availability and adequacy of food within a community or region” [19] and comprises three sub-environments: community, organizational and consumer. The community food environment (number, type, location and accessibility of retail food outlets); the organizational food environment (type and availability of healthy food within settings, such as workplaces, schools and at home); and the consumer food environment (price, promotion, placement, nutrition information, quality and availability of healthy food within retail food outlets) [19,20].

Importantly, food environment research should be considered alongside the concept of food insecurity, where people are unable to obtain a nutritious diet through socially acceptable means on a regular basis [21]. Within rural Australia, causes of food insecurity are discussed within five domains, these are: 1. access (economic and physical access to food), 2. inadequate supply (availability), 3. affordability, 4. inappropriate use of food (food safety, food preparation, nutritional status) and 5. trade policy [9]. Within this paper, we concentrate on three of these domains: access, supply/availability and affordability of food insecurity. The NEMS-S and NEMS-R tools are designed to collect data on food availability and access and do not aim to collect data on available food relief services, such as food pantries and community meals programs. The focus of this study is on the food and beverages stocked within retail food outlets in the area with a view to understanding access and availability of quality food produce at an affordable price. Whilst it might be expected that food insecurity would be linked with under-weight, research indicates that obesity is most prevalent amongst those at highest risk of food insecurity [22,23]. Other broad reaching health effects that have been identified in the literature include, disturbed sleep patterns, maternal depression, type 2 diabetes, anaemia poorer child health and higher rates of hospitalisation linked with poor infant feeding practices. Lifelong impacts include learning difficulties and adverse developmental outcomes [3].

These two major public health issues of obesity and food security should therefore be considered simultaneously, using local food environment data to inform positive environmental changes to enhance health.

Food environment interventions have the potential to improve population level diet quality in an equitable manner by ensuring an affordable, high quality food supply [24], and Glanz et al. argue that community and consumer food sub-environments should be given particular attention as they have the potential to promote and impact healthier choices at the point of purchase [20]. Measurement of community and consumer food environments is problematic, however, a recent review of retail food environment measures has provided some direction [25] by identifying the most common store types as supermarkets, grocery stores, convenience and corner stores. These store types are commonly categorized by number of registers [26,27] or sum of aisle length [28]. The review [25] also identified the two most frequently internationally used measures as the USDA’s Thrifty Food Plan tool, developed to identify food and beverage purchases to meet minimum USA healthy diet requirements [29]; and the Nutrition Environment Measurement Survey for Stores (NEMS-S) which assesses the nutrition environment more broadly based on availability, price and quality [30]. NEMS-S is one of a suite of nutrition environment measurement tools that have been assessed for interrater reliability, test-re-test reliability and face and criterion validity; and adapted for use in several studies [30]. The NEMS-R tool comprises an observational checklist of 25 items [31] and is designed to assess the availability of healthier food and beverages on main and children’s menus. In Australia, studies of food environments have focused on availability and access in urban settings and remote Indigenous communities, with most exploring food pricing [32].

Where nutrition environment measurement tools have been used in rural settings, either internationally or in Australia, they have presented with limitations to efficacy in these settings. For example, studies of food pricing commonly rely on a ‘healthy food basket’ conceptualization [33], where data include prices of a pre-defined list of ‘healthy’ foods in quantities representative of various household units, thereby enabling comparison across regions and over time [12]. However, exclusion of generic brands [34], exclusion of stores that contain fewer than 90% of the 44 items, and lack of quality assessment of fresh produce limit its usefulness and applicability in rural and remote areas.

While the definition of food environments is clear, and there is growing awareness of the need to intervene, less is known about the true disparity in the healthfulness of food environments in rural compared to urban areas. There is a paucity of evidence of the quality of rural food environments generally [15] and in Australian non-Indigenous communities specifically. The aim of this paper is to describe the food environment in a rural Australian community for use in future development of community-relevant food supply interventions.

## 2. Methods

### 2.1. Design

Census audit of rural food environment using the NEMS-S [30] and NEMS-R tools [31].

### 2.2. Context

This study took place in a rural, remote local government area (LGA) within Australia, as part of a broader community-wide obesity prevention study. Within the study, community stakeholders identified the local food supply as a determinant of unhealthy weight. Data published in 2014 show the LGA experienced a very poor chronic disease risk profile and above average adult prevalence of overweight and obesity at around 15% above the state average at that time. Concomitant health behaviours were of concern with high sugar sweetened beverage per capita consumption almost twice the state average and higher than average take-away meal consumption [35]. Located 350 km from the nearest capital city, the LGA has a total population of approximately 7000 people spread across approximately 7000 km^2^ comprising farming land and several rural and remote towns with populations ranging from between 150 and 2302 people. The predominant crops include various grains and legumes which are be ‘shipped out’ for processing [36]. Within Australia there are four major chain supermarkets, and the smallest of these has a presence within this community.

### 2.3. Selection of Retail Food Outlets

We used the categories in Table 1 to define retail food outlets. These were adapted to the rural Australian context from Glanz et al. [20] and Innes-Hughes [26].

Thirty-nine retail food outlets were identified across the LGA using the community directory available on the LGA website. ‘Ground truthing’ (physically viewing and recording of outlets) identified that an additional four outlets had opened, and that three outlets had closed. Two petrol stations were excluded, as their food supply was extremely limited. All food outlets operating at the time of the study that met the definitions outlined in Table 1 were included (n = 38).

### 2.4. Selection of Food Environment Measurement Tools

We used the Nutrition Environment Measures Survey for Stores (NEMS-S) and Restaurants (NEMS-R) due to their validated status and peer-reviewed evidence of use in a variety of settings. NEMS-S scored high on reliability; percent agreement (92–100%), inter-rater reliability kappas (0.84–1.00) and test-retest (0.73–1.00) [30]. NEMS-R also scored generally high on inter-rater reliability, kappas mostly greater than 0.80 (0.27–0.97); percent agreement (77.6–99.5%), and test-retest: most kappa values greater than 0.80 (0.46–1.0) [31]. Scoring of the NEMS-S tool was based on the published scoring tool [30] and NEMS-R was scored using the revised scoring system provided in 2011 [37].

### 2.5. Nutrition Environment Measures Survey for Stores (NEMS-S)

The original, American-based, NEMS-S tool [30] includes 11 indicator food categories: milk, fruit, vegetables, ground/minced beef, hot dogs, frozen dinners, baked goods, beverages—diet soft drink and fruit juice, bread, chips, breakfast cereal. These food categories reflected the fat and calories of a typical diet and those most recommended for healthful eating at the time the tool was developed (2007) [30]. The following modifications were made to the NEMS-S tool for the Australian context. Measures were converted from imperial to metric, and chicken (skin on and skin off) was substituted for hotdogs (being a more commonly available and consumed food in Australia). Australian reference brands were used, and Australian seasonal fruit and vegetables were included. Common breakfast cereal and bakery options were also included. Due to the importance of calcium rich foods in the Australian Guide to Healthy Eating [38], data were collected on the availability and price comparisons of cheese and yoghurt, however, these were not included in the NEMS scoring protocol to allow comparison with previous studies.

The modified NEMS-S tool was piloted in three rural community retail food outlets to test for face validity. Our modified tool maintained the 11 categories as per the NEMS-S scoring tool with a maximum possible score of 54, (availability: maximum 30, pricing: maximum 18 and quality: maximum 6 (as fruit and vegetables only included)). Between one and three points were awarded for availability depending on product type and the number of varieties available. Affordability is assessed through comparative pricing. The price of food was scored comparatively with two points being awarded if the price of better nutrient profile food was cheaper than the regular varieties. Quality was scored as ‘acceptable’ or ‘unacceptable’ based on the appearance of the majority of a given type of fruits or vegetables; an unacceptable rating was applied if the produce was ‘clearly bruised, old looking, over-ripe, or spotted’ [30] (p. 284). As per the NEMS scoring protocol, a quality score of 1 was awarded if 25–49% of the produce met an acceptable standard, 2 points were awarded if between 50–74% of the produce was acceptable and 3 points awarded if 75% or more of the produce was ‘acceptable’. An overall score combining all three dimensions was calculated. A higher score obtained in the NEMS-S tool equates to a healthier food environment.

### 2.6. Nutrition Environment Measures Survey for Restaurants (NEMS-R)

NEMS-R comprises a menu review and observational visit, (and if required, an interview with restaurant staff) to review the 25 items assessed for availability of healthier food and beverages on menus. Points are awarded for healthier options, for example: low fat dressings, whole grain breads, baked rather than fried foods, among others [31]. Factors that support or challenge healthy eating are measured and further points are awarded for signage/promotions, nutrition information and notations on menus. Points are deducted for unhealthy promotions such as super-sizing, all-you-can-eat offers, and unhealthy combo-meal deals [31]. Possible NEMS-R scores range from −5 to 23 (for establishments without a specific children’s menu) and −8 to 32 (for establishments with a children’s menu) [39].

Minor modifications to language were required to ensure NEMS-R was relevant to the Australian context. The term ‘entree’ in USA generally means main course and in Australia it means a smaller first course, therefore all references to entrée were removed and replaced with the Australian language of ‘main course’. The size of the meal was captured through the retention of the question related to reduced-size portions offered on menu. NEMS-R collects data on ‘low carb promotions’ which was the ‘diet fad’ of choice at the time the tool was developed (2007). Instead we collected data on any ‘diet fads’ outside the Australian Guide to Healthy Eating (AGHE), such as raw foods or paleo diets that were popular at the time of data collection (2017). We also modified milk to include all lower fat milk options, typically 2% fat milk in Australia, (NEMS: 1% or non-fat). We administered the NEMS-R tool on all food service outlets in the study LGA. As there are no fast food chain outlets in the study LGA to enable a comparison between small family owned businesses and large chain store food outlets we administered the NEMS-R on a neighbouring large fast-food chain outlet. As per NEMS-S we pilot tested the modified NEMS-R for face validity. A higher score on NEMS-R also indicates a healthier food environment.

### 2.7. Data Collection

Prior to data collection, JW and PL undertook online NEMS training [40], then provided face-to-face training to CR and FG. Data were collected over four days from 19 to 22 June 2017. Retail food outlets were not informed of the assessment ahead of time, permission was obtained on entering the premises. Store owners were advised of the purpose of the study on entering the premises. Most of the data could be collected without interaction with the staff of the food premises. Where interaction was required, food retailers freely shared the required information.

All researchers conducted an initial NEMS-S and NEMS-R survey together and thereafter worked in pairs to ensure consistency between scoring. Test-retest reliability was performed on a 5% sample (n = 2/38) as per NEMS protocol. The results indicated a high level of test-retest reliability with NEMS-R kappa of 0.825 and NEMS-S kappa of 0.781. Surveys took between 20 and 60 min to complete dependent on the size of the outlet. Data were recorded using hard copy survey sheets and then entered into the relevant NEMS-S or NEMS-R Excel spreadsheet. A random sample of 25% of data entries was assessed for accuracy. No errors were found in this cross check. Ethical approval for this study was obtained through Deakin University [HEAG-H 80_2016].

### 2.8. Data Analysis

Data were prepared using NEMS Excel spreadsheets and the NEMS scoring system [30,37,39] and STATA release 15 [41]. Primary outcome measures were: availability, price and quality of healthy foods compared across store types (supermarkets and general stores), food service (restaurants, fast casual—hotels, fast casual—cafes, fast food), and geographic locations to identify if cost, availability, promotions, healthiness and quality of food varied across the LGA. Food was classified into the core food groups of the Australian Guide to Healthy Eating to determine if the foods recommended were available and if they were more or less expensive than unhealthier foods. Descriptive statistics on the availability of the Australian core food groups and discretionary foods were reported. For NEMS-S data, *t*-tests were used to compare the availability, price, quality of food between type of store (supermarket or general store) and separate linear regressions were used to compare the availability, price, quality between communities (north, central, south). For all statistics, *p*-values < 0.05 were deemed statistically significant.

Published studies appear to have applied different scoring protocols, therefore, we have converted our NEMS scores to percentage figures to enable comparison with other studies using these tools. In keeping with NEMS tools a higher percentage indicates a food environment more conducive to healthy eating.

## 3. Results

The exploration of the community food environment found a total of 38 outlets, all of which are included in the data analysis (100% RR), with 11 being food stores and 27 food service outlets (sit-down n = 13, fast-casual n = 7, fast food n = 7).

### 3.1. Food Stores

Of the 11 food stores, five were supermarkets and six were general stores. Table 2 shows the maximum possible score and mean scores for each of the three sub-categories of availability, price and quality for stores overall and by type of food store (the higher the score, the healthier the food environment). A total mean score of 21.0 (SD 4.6) out of a possible 54 points was obtained for the LGA as a whole. The overall NEM-S score was significantly higher for supermarkets (mean = 24.8, SD 2.6) than general stores (mean = 17.8 SD 3.2; t(2,9) = 3.9, *p* < 0.05) as was the availability of healthy foods score (supermarkets (mean = 21.4, SD 3.0); general stores (mean = 12.0, SD 3.0; t(2,9) = 5.2, *p* < 0.05).

Table 3 compares the mean NEMS-S scores for stores in the north (n = 3), central (n = 5) and south (n = 3) of the LGA. The mean NEMS-S score was highest in the central area at 24.8 (44%) where the two largest supermarkets were located. There were no statistically significant differences on any category between areas.

A breakdown of the availability of healthier choices and price differential across the Australian core food groups is shown in Appendix A. In most, but not all cases regardless of store types, healthier choices were more expensive and less available.

All supermarkets and general stores sold reduced fat milk. The price comparisons showed the price of reduced fat milk was more expensive than full fat milk in all stores. Low fat cheese and/or low fat yoghurt was only available in 27% of food stores and, where it was available’ low fat options were more expensive than full fat. Wholegrain bread was available in all stores and more expensive than white bread in 27% of stores. Overall, healthier varieties of cereals (including rice, pasta, grains) were more expensive 60% of the time compared to their healthier counterparts. Low fat minced beef (ground beef) was available at two food stores across the Shire; at one of these it was more expensive than full fat minced beef and the other store stocked only low-fat minced beef so no price comparison was possible. The remaining nine stores stocked full fat varieties only. All five supermarkets and four of the six general stores stocked a wide variety of fruit and vegetables (10 or more). None of the food stores displayed any healthy eating promotions at the time of the surveys.

### 3.2. Food Service Outlets

Twenty-eight food service outlets were surveyed however, one café closed before collection of follow-up information and was excluded from the study (n = 27). All food service outlets were independent stores, with no fast food chain outlets in this rural community. Table 4 describes the type of food service establishment, number of outlets, explanation of the categorization, the mean and standard deviation NEMS-R score.

The mean NEMS-R score was low at 3 (SD 4.0) out of a possible 23, (excluding children’s menu) [39] across the LGA. Nine of the 27 food service outlets offered a children’s menu, eight of these scored two points, one scored five points (possible scoring range −3 to 9). One fast food outlet received a negative score indicating that the store sold almost exclusively unhealthy foods and encouraged over-consumption by promoting up-selling through ‘meals deals’ that comprise fried foods and sugar sweetened beverages at a price cheaper than purchasing items individually. Aside from one café in the central area scoring 17, no other food service outlet scored higher than eight. NEMS-R scoring of a comparison neighbouring fast food chain store, that predominately sold fried food, scored 10. Table 5 shows comparisons between different areas of the LGA, with the central area having a slightly better food environment as scored on NEMS-R.

There was no statistically significant difference between these geographic boundaries or between types of outlets and the mean total. Table 6 reports NEMS-R according to food service outlet and compares means across the three measures of availability, facilitators and barriers to healthy eating. Means scores across hotels, cafes, fast food and bakeries were all low (maximum possible scores, means and standard deviations shown in the table). There were no statistically significant differences between these store types, though café’s in general scored higher than hotels/pubs. Health promoting practices, as defined by NEMS-R, include signage/promotions, nutrition information/notations on menus, and reduced portion sizes.

The most frequent practice observed was the provision of diet soda (100% of food service outlets), and low fat milk (about two thirds of food service outlets) with two using it as the default option in hot and cold drinks. Across the area, 23% of food service outlets offered at least one main meal designated as ‘healthy’ according to NEMS-R standards. Fried, french fry-style potato chips were served at the majority (88%) of food service outlets. About a third (37%) offered a children’s menu, from which one menu item met the NEMS-R definition of ‘healthy’ and two menu items included a healthy side as per published definition [31]. About a quarter (23%) offered unprocessed fruit for sale, 15% had non-fried vegetables identified on their menus. Across all food service outlets, just under half had wholemeal bread available, and just over half offered 100% fruit juice. Nutritional information and healthy menu items were identified in only one food service outlet. Bottled water was available for sale at all food service outlets, with some offering free tap water. Data on the collection of freely available tap water was not a component of the NEMS-R tool.

## 4. Discussion

The aim of this study was to describe the food environment in a rural Australian community in order to inform future community-relevant food supply interventions. We provide a comprehensive account of the community and consumer food environments in this LGA. The findings provide evidence that major changes to the food environment are needed for healthy foods to be available equitably to all community members. Food stores scored poorly on food availability and comparative pricing. Among food stores, healthier options were more expensive than their unhealthy alternative, and we observed variable quality of fresh fruit and vegetables. The availability of food service outlets (n = 27) was more predominant than food stores (n = 11), with the majority (n = 26) receiving low scores indicating healthy choices of prepared food were generally difficult to obtain across this LGA.

While NEMS tools have been used internationally across a variety of settings including rural environments [42,43], many have adapted the tool to local context thereby limiting direct comparability of these findings with our study. There are also no similar studies within Australia either measuring the food environment of a whole rural LGA or using the NEMS tools to undertake a comprehensive food environment audit.

### 4.1. Food Stores

Generally smaller store sizes have been correlated with lower NEMS-S scores and fewer healthy foods than larger supermarkets [44]. In our study, there was no statistically significant difference in the comparative pricing score between supermarkets and general stores, this may be due to a number of reasons. Firstly, both scores for comparative pricing (healthy vs. unhealthy price of a similar product), were very poor (1.2 and 1.0 respectively out of a possible 18 points); secondly, the small number of stores across the LGA limits statistical analysis, thirdly all supermarkets were small in size with the largest one having five registers, the smallest just one register, this limits the stock they can carry and may constrain their bargaining power in regards to food supply logistics to obtain healthy choices at a reasonable price. We consider the lack of variability in the quality score to be related to the quality scoring systems within the NEMS protocol where an 85% score translates to 100% on analysis. We consider some of our stores were over-scored on quality due to this protocol.

Our findings are consistent with studies that have identified rural areas typically have smaller and fewer supermarkets which equates to less variety, poorer quality and higher prices than in urban areas [7,43,45]. In Australia, four major supermarket chains exist, only the smallest of these was present in this community, all food stores in this LGA would be considered small in an urban context. In comparison with international studies, our food store environment score (39%) is lower than scores obtained in rural USA (around 60%) [7,42]. Our very low score indicates a food environment that is not conducive to healthy eating. However it also provides a baseline environment score and potential opportunity for food supply interventions to make a big impact on the availability of healthy choices.

Transport services to remote Indigenous communities are cited as barriers to a healthier food supply [46]. We contend that non-Indigenous rural and remote areas, locally and internationally, experience similar issues and all may benefit from food stores working together to negotiate lower freight costs [9], thereby, increasing supply and lowering prices. In the shorter term, interventions supported by food store owners show potential to improve healthier choices, these include: taste tests, free samples of healthier choices and communication interventions [47], also nutrition-style shelf labelling has been shown to be effective at nudging healthier choices [48].

Food store owners perceive that barriers to purchasing, stocking and promoting healthy food include consumer preferences for high fat, high sugar and low prices, along with lower wholesale availability of healthy food [47]. Within a small, low income, low profit-margin community, a useful strategy may be to provide financial incentives to healthy food procurement [8]. All interventions should not only focus on store proximity but availability of healthy choices [49]. Given that rural residents typically demonstrate strong loyalty to local food stores [50], these stores are well placed to positively impact dietary choices.

### 4.2. Food Service Outlets

Across the study area there are no major chain fast food outlets but 27 independent locally owned pubs, fast food outlets and cafes, with the majority of these providing inexpensive readily available high fat foods. Although the methodology differed, our findings are consistent with the Australian study by Innes-Hughes et al. (2012) [26] where take-away outlets in each town offered very few healthy foods, and high fat choices dominated menus.

Consistent with Pereira [43] we found the most widespread ‘healthier practice’ within food service outlets to be the availability of diet soda (100% in our study c.f. 80%). Healthy menu item availability was low with less than 30% of venues offering even one healthy choice (as defined by NEMS) [42,43]. The one food service outlet that scored well (74%) applies the State Government Healthy Choices Guidelines [51] supporting product promotion, placement and healthy meal deals. Other than this higher score, we observed that our comparison fast food chain outlet scored better than most of the food service outlets within the LGA, mainly due to the signage used rather than the health of the food on offer.

Our very low baseline restaurant score of 3 (SD 4.0) is indicative of an urgent need to intervene with multiple opportunities to improve the healthiness of food offerings within food service outlets [50]. Martinez-Donate (2015) [42] reported improved NEMS-R scores post introduction of a suite of strategies to promote healthier choices, including point-of-purchase labelling and promotions of healthier items. Children’s menus could be improved through the introduction of healthy sides as default and reducing serving sizes [52]. Changing to healthier oils in takeaway outlets has been shown to reduce saturated fat intake in previous studies [53] and given the pervasiveness of deep fried food in the study area, a reduction in the use of unhealthy fats may be a useful first step in conjunction with the broader systemic changes required.

### 4.3. NEMS Tools for a Rural Australian Context

We identified limitations with the NEMS tools in the Australian rural context. With regards to NEMS-S, we considered the quality score protocol often created an over-statement of actual produce quality. Where we assigned a score of 75%, this was equated to 100% score in the overall score. In regards to NEMS-R we considered a smaller portion at a cheaper price should have been awarded a score. We also consider ready access to free tap water should receive additional points. We have some concerns about the importance placed on nutritional promotion given the example of our comparison store, which scored better than most stores, despite the mainly unhealthy food offerings.

### 4.4. Strengths

To our knowledge, this study is the first in Australia to apply the validated NEMS tools including all food stores across a single local government area, and is one of very few examining the food environment in a rural Australian context. Our study used ground-truthing to provide an accurate representation of the current food environment at a given point in time.

### 4.5. Limitations

By only collecting data within the LGA geographical borders, out-shopping to larger towns has not been accounted for. In this study there was no comparison group. The study was conducted at a single point in time so does not necessarily provide data on usual food availability, quality or pricing. One might expect seasonality would contribute to variation of produce, or variations in days of the week, which may be influenced by supplier drop off or residents’ weekly shops. This study was limited to the rural, Australian context in which it was conducted, thus application of the results may not be appropriate for or applicable to other contexts. Also food environments can change rapidly, as evidenced in this and similar studies by businesses closing during the time of the audit [7].

## 5. Conclusions

Our findings showed that the healthfulness of the food environment for this remote local government area of rural Victoria is poor. Healthy options, nutrition information and nutrition promotion are not available at most food stores across the LGA.

Outside a supermarket it is very difficult to purchase healthy food in this rural community, made more challenging by the fact that supermarkets are often a significant distance from residents’ homes. Given that food environments are a key determinant of obesity [54] and rural loyalty to local business [49], the current predominately unhealthy food environment provides scope to work with food retailers and consumers to ensure healthier options are more visible, available and affordable. This may enable consumers to make healthier choices and thereby impact positively on the health of this community.

Successful interventions have utilised multipronged strategies to improve both food supply and customer demand [8]. We consider it a priority to affect the food environment as a frontline intervention before embarking on, or alongside, any individual behaviour change strategies to ensure healthy food choices are available when consumers seek to choose these.

## 6. Recommendations

More research is required to explore the relationship between the food environment and food security in rural Australia and with health of those who reside there.

## Figures and Tables

**Table 1:** Categorization of retail food stores and food service outlets.

Food Stores	Food Service
Supermarket—sells food products and other items, large scale, may open for extended hours on most days of the week. (Register numbers: 1 to 5)	Restaurants Sit-down—order and pay at table, table service, food eaten at outlet e.g.,: traditional restaurants
General—sells food products and other items, small scale, typically in a small town. General stores generally have reduced hours and usually are not open on weekends.	Fast-casual—order and pay at counter, may have table service, food eaten at outlet or taken away e.g.,: hotels (pubs) and cafés
Convenience stores (North American)—extended hours, stocking a limited range of household goods and groceries.	Fast-food—order, pay and served food at counter, quick service, food usually eaten away from outlet e.g.,: take-aways and bakeries

**Table 2:** NEMS-S Food Stores Scores for all stores, grocery stores and general stores and *p* values resulting from *t*-tests between store type.

		All Food Stores (n = 11)		Supermarkets (n = 5)		General Stores (n = 6)		*p*
	Max	Mean	SD	Mean	SD	Mean	SD	
Availability	30	16.3	5.7	21.4	3.0	12.0	3.0	*p* < 0.05
Price	18	1.1	1.0	1.2	1.1	1.0	1.1	NS *
Quality	6	4.6	2.1	5.4	1.3	4.0	2.5	NS *
Total Score	54	21.0	4.6	24.8	2.6	17.8	3.2	*p* < 0.05

* NS—Non-significant at *p* ≥ 0.05.

**Table 3:** NEMS-S score means from stores in the northern, central, and southern areas of the rural local government area.

Food Stores (n = 11)		North (n = 3)		Central (n = 5)		South (n = 3)	
	Max	Mean	SD	Mean	SD	Mean	SD
Availability	30	14.0	5.2	19.0	8.7	17.3	2.5
Price	18	1.2	1.1	1.3	1.2	0.7	1.2
Quality	6	4.8	1.6	6.0	0.0	3.0	3.0
Total Score	54	20.0	4.4	24.0	4.6	19.7	5.1

**Table 4:** Types and number of food service outlets across the Local Government Area and a comparison fast food chain outlet with their mean NEMS-R Scores and their scores as a percentage of the maximum NEMS-R Score (−5 to 23).

Food Service	Number of Outlets	NEMS-R SCORE		% of Max NEMS-R SCORE (−5 to 23)
	N	Mean	SD	%
Fast Casual: Hotels/pubs/restaurant	13	1.8	1.6	7.8
Fast Casual: Café	7	7.0	5.6	30.0
Fast Food: Take-aways	5	2.0	2.9	8.7
Fast Food: Bakeries	2	4.0	0.7	17.4
Total	27	3.0	4.0	13.0
Comparison	1	10.0	0.0	43.0

**Table 5:** NEMS-R score means for the north, central and south of the Local Government Area.

	Score Range	NORTH			CENTRAL			SOUTH		
Number of outlets	27	9			13			5		
		MEAN	SD	% *	MEAN	SD	%	MEAN	SD	%
NEMS-R score	−8–32	2.0	1.0	6%	4.0	5.0	12%	3.0	3.0	9%

* percentage score calculated as a % mean of maximum possible score.

**Table 6:** NEMS-R scores by type of food service outlet and health promoting practices.

Type of Outlet		Total		Hotels/Pubs/Restaurant *		Cafes		Fast Food		Bakeries	
Outlets (n (%))		27 (100)		13 (48.1)		7 (25.9)		5 (18.5)		2 (7.4)	
NEMS-R items	Possible Score (A score closer to the maximum possible score indicates a healthier food environment.)	M	SD	M	SD	M	SD	M	SD	M	SD
Availability of healthy choices	0–15	2.8	2.5	1.4	1.4	5.3	3.1	2.6	1.5	3.5	0.7
Facilitators of healthy eating	0–8	0.4	1.4	0.2	0.4	1.1	2.6	−1.0	1.4	0.0	0.0
Barriers to healthy eating	−5–0	−0.3	0.8	0.0	0.0	−0.3	0.8	−1.0	1.4	0.0	0.0

* The scores of the one restaurant were combined with hotels/pubs to preserve anonymity.

## Appendix A

**Table A1:** Availability of more healthful options, and pricing features for supermarkets and general stores, across the local government area, 2017.

Core Food Group (AGHE ref)	Total N = 11	Super-Markets N = 5	General Stores N = 6
**Availability of healthier breakfast cereal > 2 varieties**	9	5	4
n (%) cost healthy cereal < unhealthy	5	3	2
**Whole grain bread availability**	10	5	5
>2 varieties whole wheat bread	8	5	3
Price same for both	7	4	3
Price higher for whole wheat	3	1	2
**DAIRY OR ALTERNATIVES**			
Low-fat/skim **milk** available	11	5	6
Price Higher for low-fat/skim milk	11	5	6
Low-fat **cheese** available	3	3	0
Price Same for both	2	2	0
Price Higher for low-fat	1	1	
**Low fat Yoghurt availability**	3	3	0
Price Lower for lowest-fat	1	1	0
Price Same for both	1	1	0
Price Higher for low-fat	1	1	
**FRESH FRUIT AVAILABILITY—NUMBER OF VARIETIES**			
<5 varieties	1		1
5–9 varieties	3		3
10 varieties	7	5	2
**FRESH VEGETABLES AVAILABILITY—NUMBER OF VARIETIES**			
5–9 varieties	2	0	2
10 varieties	9	5	4
**MEAT OR MEAT ALTERNATIVES**			
**Low-fat mince availability (beef or turkey)**	2	2	0
Higher price for lean meat	1	1	
**Chicken availability—skinless breast**	5	4	1
Price Higher for skinless	3	3	
Legumes available (could also be classified as vegetables)	10	5	5
Eggs available	10	5	5
**DISCRETIONARY FOODS**			
**Healthier snack alternatives to chips (e.g., Grain Waves) (**Baked alternative to fried potato crisps**)**	4	4	0
**Chips- Price- (**Fried potato crisps**)**			
Price Lower for Grain Waves than Smiths Chips	0	0	0
Price Higher for Grain Waves than Smiths Chips	4	4	
**Healthier dry biscuits available (e.g., water crackers)**	11	5	6
Price Lower for water crackers than BBQ shapes (Savoury crackers)	10	5	5
**Diet soft drinks available**	10	5	5
Same price for both	10	5	5
**100% Juice Availability-**	9	5	4
Lower for 100% juice (2 L)	1	1	
Same for both	1		1
Higher for 100% juice (2 L)	4	2	2
